# Inflammation and Cancer: Role of Annexin A1 and FPR2/ALX in Proliferation and Metastasis in Human Laryngeal Squamous Cell Carcinoma

**DOI:** 10.1371/journal.pone.0111317

**Published:** 2014-12-09

**Authors:** Thaís Santana Gastardelo, Bianca Rodrigues Cunha, Luís Sérgio Raposo, José Victor Maniglia, Patrícia Maluf Cury, Flávia Cristina Rodrigues Lisoni, Eloiza Helena Tajara, Sonia Maria Oliani

**Affiliations:** 1 From the Post-graduation in Structural and Functional Biology, Federal University of São Paulo (UNIFESP), Paulista School of Medicine (EPM), São Paulo, SP, Brazil; 2 Department of Molecular Biology, Faculty of Medicine (FAMERP), São José do Rio Preto, SP, Brazil; 3 Department of Otorhinolaringology, Faculty of Medicine (FAMERP), São José do Rio Preto, SP, Brazil; 4 Department of Pathology, Faculty of Medicine (FAMERP), São José do Rio Preto, SP, Brazil; 5 Department of Biology and Zootechny, São Paulo State University (UNESP), Ilha Solteira, SP, Brazil; 6 Department of Genetics and Evolutionary Biology, Institute of Biosciences, University of São Paulo (USP), São Paulo, SP, Brazil; 7 Department of Biology, Instituto de Biociências, Letras e Ciências Exatas (IBILCE), São Paulo State University (UNESP), São José do Rio Preto, SP, Brazil; Virginia Commonwealth University, United States of America

## Abstract

The anti-inflammatory protein annexin A1 (ANXA1) has been associated with cancer progression and metastasis, suggesting its role in regulating tumor cell proliferation. We investigated the mechanism of ANXA1 interaction with formylated peptide receptor 2 (FPR2/ALX) in control, peritumoral and tumor larynx tissue samples from 20 patients, to quantitate the neutrophils and mast cells, and to evaluate the protein expression and co-localization of ANXA1/FPR2 in these inflammatory cells and laryngeal squamous cells by immunocytochemistry. In addition, we performed in vitro experiments to further investigate the functional role of ANXA1/FPR2 in the proliferation and metastasis of Hep-2 cells, a cell line from larynx epidermoid carcinoma, after treatment with ANXA1_2–26_ (annexin A1 N-terminal-derived peptide), Boc2 (antagonist of FPR) and/or dexamethasone. Under these treatments, the level of Hep-2 cell proliferation, pro-inflammatory cytokines, ANXA1/FPR2 co-localization, and the prostaglandin signalling were analyzed using ELISA, immunocytochemistry and real-time PCR. An influx of neutrophils and degranulated mast cells was detected in tumor samples. In these inflammatory cells of peritumoral and tumor samples, ANXA1/FPR2 expression was markedly exacerbated, however, in laryngeal carcinoma cells, this expression was down-regulated. ANXA1_2–26_ treatment reduced the proliferation of the Hep-2 cells, an effect that was blocked by Boc2, and up-regulated ANXA1/FPR2 expression. ANXA1_2–26_ treatment also reduced the levels of pro-inflammatory cytokines and affected the expression of metalloproteinases and EP receptors, which are involved in the prostaglandin signalling. Overall, this study identified potential roles for the molecular mechanism of the ANXA1/FPR2 interaction in laryngeal cancer, including its relationship with the prostaglandin pathway, providing promising starting points for future research. ANXA1 may contribute to the regulation of tumor growth and metastasis through paracrine mechanisms that are mediated by FPR2/ALX. These data may lead to new biological targets for therapeutic intervention in human laryngeal cancer.

## Introduction

Laryngeal cancer is one of the most common types of head and neck tumors that has a high mortality rate and a poor prognosis [1]. More than 12,500 new cases of laryngeal cancer are diagnosed annually and 3,560 annual deaths occur [2]. The development of better treatment as well as better diagnostic and preventive approaches requires an improved understanding of the complex process of laryngeal tumorigenesis.

Only 5% to 10% of all cancers are caused by the inheritance of mutated genes, whereas the remaining 90% to 95% of cases have been linked to genetic and epigenetic alterations caused by lifestyle and environmental factors, such as cigarette smoking and alcohol use [3], [4]. It is now well recognized that inflammation is a risk factor for most types of cancer, including laryngeal carcinomas [5], [6]. Chronic inflammation has been linked to various steps involved in tumorigenesis, including cellular transformation, promotion, proliferation, invasion, angiogenesis, and metastasis [7], [8]. Hanahan and Weinberg, in their recent review [9], recognized inflammation as a new hallmark of cancer that promotes multiple tumor features.

Inflammatory cells secrete numerous cytokines, chemokines and growth factors that can stimulate proliferation, inhibit apoptosis, induce morphogenesis and generate DNA-damaging reactive oxygen species [7], facilitating genomic instability [10]. Furthermore, these cells synthesize vascular endothelial growth factor (VEGF), angiopoetin, metalloproteinases and other proteins that can stimulate vascular endothelial cell mitosis and extracellular matrix remodeling [11]. Therefore, inflammation generates not only changes in the microenvironment to promote cancer but also changes that stimulate neoplastic progression.

It has been demonstrated that mast cells and neutrophils can be recruited by tumor cells. Increased numbers of mast cells have been reported in mammary [12] and lung carcinomas [13], among other tumors. Experimental evidence has indicated that activated mast cells release angiogenic growth factors and regulators, prostaglandin, metalloproteinases and cytokines [14], and may play a dual role in promoting or inhibiting tumor growth depending on the local stromal conditions [15], [16]. Of the cell types residing in the tumor microenvironment, tumor-associated neutrophils (TANs) have received little attention [17]. Although there is evidence suggesting a role for neutrophils in enhanced disease progression in specific human tumors, the relationship between neutrophil infiltration and prognosis in human cancer has not been systematically investigated [18]. The role of neutrophils in tumor progression remains controversial [19] largely because these cells have both tumor-promoting and tumoricidal functions depending on the presence of transforming growth factor beta (TGF-β) [20], [21].

From these studies, it is clear that various inflammatory mediators are differentially expressed in several cancers and can stimulate disease progression [22]. Thus, agents that suppress these inflammatory mediators represent potential targets for the treatment of cancer. The anti-inflammatory protein annexin 1 (ANXA1), a 37-kDa member of the annexin superfamily, is a steroid-regulated protein that is implicated in mediating some of the beneficial activities of glucocorticoids [23], including the regulation of cell proliferation [24], differentiation [25] and metastasis [26].

Dysregulation of ANXA1 has been reported in multiple neoplasms, suggesting that this protein may play important roles in tumor development and progression [27]. ANXA1 is overexpressed in breast cancer [28] and hepatocellular carcinoma [29] but is markedly down-regulated in esophageal [30], [31] and head and neck carcinomas [32]. However, the molecular mechanisms by which ANXA1 modulates cellular responses have not been fully determined.

Advances in this area have shown a functional link between the anti-inflammatory properties of ANXA1 and receptor for formyl-Met-Leu-Phe (fMLP) FPR [33], a specific class of G protein-coupled 7-transmembrane receptors [34]. In humans, three family members have been identified, FPR1, FPR2/ALX (FPRL1) and FPR3 (FPRL2) [35]. Some of the effects of exogenous ANXA1 can be attenuated by Boc2, an antagonist of both FPR1 and FPR2 [33]. Recently, it has been shown that the ANXA1 N-terminal peptide, ANXA1_2–26_, promotes the migration of WS1 human skin fibroblasts [36], and the invasiveness of SKCO-15 colorectal adenocarcinoma cells [24] through interaction with FPRs. However, no data are available regarding the role of ANXA1 and FPRs in laryngeal squamous cell carcinoma.

Several signaling pathways mediate inflammation-associated tumorigenesis. For example, cyclooxygenase-2 (COX-2), an enzyme involved in the conversion of arachidonic acid to prostaglandins, is considered to play important roles in cancer development by modulating cell proliferation and other important biological processes via their metabolites, G protein-coupled receptors and downstream effectors [37]. COX-2 is up-regulated in several malignancies [38]–[40], including head and neck carcinomas [41]. Actually, Abrahao et al. (2010) have shown that the pro-inflammatory COX-2 metabolite prostaglandin E2 and its EP3 receptor can activate head and neck carcinoma cells to proliferate and is a potential target for preventive approaches. Considering that glucocorticoids are highly effective inhibitors of COX-2 expression [42], the steroid-regulated protein annexin A1, which is implicated in mediating some of the activities of glucocorticoids, may also be involved in the inflammatory network linked to COX-2.

Given the potential role for ANXA1 in multiple neoplasms such as the regulation of cell proliferation, differentiation and metastasis, we focused on the molecular mechanisms by which ANXA1 modulates these cellular responses. In this report, we showed that ANXA1 protein is down-regulated in human laryngeal carcinoma and can regulate tumor growth in a paracrine manner that is mediated by the receptor FPR2/ALX. This investigation demonstrates the potential significance of this ANXA1/FPR2 interaction in tumorigenesis. Overall, this study identified potential roles for the molecular mechanism of this protein interaction in laryngeal cancer, including its relationship with the prostaglandin pathway, providing evidence for the potential of ANXA1 as a therapeutic target and promising starting points for future research.

## Materials and Methods

### Tissue Samples

Human invasive laryngeal cancer tissues were obtained from 20 patients with laryngeal squamous cell carcinoma who were treated at the Hospital de Base in São José do Rio Preto, Brazil. The patients were male, alcoholic smokers, their age ranged from 50 to 80 years, and none of them had received radiotherapy or chemotherapy before intervention. In each patient (n = 20), the surgical tumor samples were collected from the center of the laryngeal tumor, the peritumoral samples were collected from the tumor periphery, and the control samples were collected from the region free of tumor cells. Because of the high heterogeneity of laryngeal carcinoma, the tumor tissues were dissected and reviewed by a senior pathologist and were characterized as moderately differentiated, invasive carcinomas. These tissue samples were used in a previous study that was conducted in our laboratory, and the number of patients was sufficient to obtain statistically significant data [32]. The study protocol was approved by the Committee of Ethics in Research of the Federal University of São Paulo, School of Medicine, Brazil (CEP-0300/08), and written informed consent was obtained from all of the patients involved.

### Cell Culture

The Hep-2 cell line, which was originally established from an epidermoid carcinoma of the larynx, was used in the present study (ATCC, Rockville, Maryland, USA). Cell line authentication was performed using the AmpFLSTR Identifiler PCR Amplification Kit (Life Technologies) at the Special Techniques Laboratory, Hospital Israelita Albert Einstein (LATE -HIAE), Sao Paulo. We found 100% identity of our cell line compared with the American Type Culture Collection (ATCC) database. The cells were seeded at a density of 2×10^6^ cells in a 75-cm^2^ culture flask (Corning, NY, USA) and incubated in MEM-Earle medium (Cultilab, Campinas, SP, Brazil), pH 7.5, supplemented with 20% fetal calf serum (Cultilab), 1% non-essential amino acids, and 0.1% antibiotic/antimycotic solution (Invitrogen Corporation, Carlsbad, CA, USA), at 37°C in a humid atmosphere of 5% CO_2_ [43].

### Drug Treatment

For the pharmacological experiments, Hep-2 cells were seeded as previously described. Twenty-four hours later, after the cells had already adhered, they were incubated in serum-free medium to synchronize the cell cycle. After an additional 24 hours, the medium was replaced with complete growth medium containing the following: (a) 1 µM of ANXA1_2–26_ peptide (Ac-AMVSEFLKQAWFIENEEQEYVQTVK) (Invitrogen); (b) 1 µM of ANXA1_2–26_ peptide and 10 µM Boc2, a nonselective FPR antagonist (N-t-BOC-MET-LEU-PHE) (MP Biomedicals, Aurora, OH); (c) 0.01 µM dexamethasone (a glucocorticoid; Sigma-Aldrich, St. Louis, MO, USA); (d) 0.01 µM dexamethasone and 10 µM Boc2; or (e) 10 µM Boc2 alone. The drugs were first dissolved in small amounts of dimethyl sulfoxide (DMSO) and then diluted in medium (the final concentration of DMSO never exceeded 1%). The concentrations of the drugs were selected based on preliminary experiments and literature data [32], [44]. The control cells were treated with complete medium with the same concentration of DMSO used to dilute the drugs. Every 2 days, the medium was replaced with fresh medium containing the drugs at the same dose; the cell morphology was observed every day [43]. After 6, 24, 48, 72 and 96 hours, control and treated cells were harvested and counted using the Countess Automated Cell Counter (Invitrogen).

### Fixation, Processing, and Embedding for Light and Electron Microscopy

Laryngeal samples and Hep-2 cells were fixed in 4% paraformaldehyde, 0.5% glutaraldehyde, and 0.1 mol/L sodium cacodylate buffer (pH 7.4) for 24 hours at 4°C, washed in sodium cacodylate, dehydrated through graded percentages of ethanol, and embedded in LRGold (London Resin Co., Reading, UK). For histopathological and morphological analyses, tissue sections (0.5-µm thick) were cut on an ultramicrotome (Reichert Ultracut; Leica, Austria), stained with 1% toluidine blue in 1% borax solution (TAAB Laboratories, Aldermaston, UK) and examined using an AXIOSKOP 2-Mot Plus ZEISS microscope (Carl Zeiss, Jena, Germany). For electron microscopy, sections (∼90 nm) were cut on an ultramicrotome and placed on nickel grids for immunogold labeling [45].

### Immunogold Labeling of Post-embedded tissues

Immunocytochemistry reaction (double labeling) was used to detect the expression and co-localization of ANXA1 and FPR2/ALX in mast cells, neutrophils, and control and tumor cells from laryngeal tissues and Hep-2 cells.

The Hep-2 cells were incubated with control medium, ANXA1_2–26_ and ANXA1_2–26_+Boc2. After 48 hours, the culture was harvested, and the cells were embedded in LRGold resin for immunocytochemical analysis. Ultrathin sections of laryngeal tissues and Hep-2 cells were incubated in a step-by-step manner using the following reagents and/or conditions at room temperature: 1) distilled water; 2) 0.1 mol/L phosphate buffer containing 1% egg albumin (PBEA); 3) 0.1 mol/L PBS containing 5% PBEA for 30 minutes; 4) sheep polyclonal antibody LCPS1, raised against the N-terminal segment of ANXA1 (1∶500 in PBEA) and rabbit polyclonal antibody FPR2/ALX (Abcam, Cambridge, UK) (1∶500 in PBEA) for 2 hours; normal sheep and rabbit sera were used as the control (1∶500); 5) three washes in PBEA containing 0.01% Tween 20; 6), donkey anti-sheep IgG (Fc fragment-specific) antibody (1∶100 in PBEA) conjugated to 15-nm colloidal gold (British Biocell, UK) was added to detect ANXA1, and goat anti-rabbit IgG (Fc fragment-specific) antibody (1∶100 in PBEA) conjugated to 10-nm colloidal gold (British Biocell) was added to detect FPR2/ALX). After 1 hour, the sections were washed in PBEA containing 0.01% Tween 20, and then in distilled water [46]. Sections were stained with uranyl acetate and lead citrate before examination on a ZEISS LEO 906 electron microscope (Carl Zeiss, Jena, Germany).

### Multiplex Assays

To quantify the pro-inflammatory cytokines interleukins 6 (IL-6) and IL-8 as well as monocyte chemotactic protein-1 (MCP-1), in the Hep-2 cell culture supernatants, we used the multiplex instrument LUMINEX xMAP MAGPIX (Millipore Corporation, Billerica, MA, USA). The cells were incubated with control medium, ANXA1_2–26_, ANXA1_2–26_+Boc2, Dexa, Dexa+Boc2 or Boc2. After 48 hours of treatment, the culture supernatants were removed, centrifuged and stored at −20°C. Antibody beads, controls, wash buffer, serum matrix and standards were prepared following the manufacturer's instructions (MILLIPLEX HCYTOMAG-60K kit). Next, 200 µL of wash buffer was added to each well of a magnetic 96-well plate and mixed on a shaker for 10 min. The wash buffer was decanted, and 25 µl of standards, controls and samples were added to the wells. Next, 25 µl of assay buffer was added to the samples, and 25 µl of serum matrix was added to the standards. Finally, 25 µl of magnetic beads (coated with a specific capture antibody) was added to all wells and incubated overnight at 4°C on a shaker. The next day, the plate was washed with wash buffer and incubated with 25 µl of detection antibodies for 1 hour on a shaker. Next, 25 µL of streptavidin-phycoerythrin was added to each well and incubated for 30 minutes on a shaker. The plate was washed and incubated with 150 µl of drive fluid for 5 minutes on a shaker. Finally, the plate was analyzed using MAGPIX with xPONENT software [47].

### Real-time PCR

The Hep-2 cells were incubated with control medium, ANXA1_2–26_ or ANXA1_2–26_+Boc2 and harvested after 48 hours. Total RNA was extracted using TRIzol Reagent (Invitrogen) according to the manufacturer's protocol. The genomic DNA was removed by DNase treatment according to the manufacturer's description (Promega). Aliquots (2 µg) of total RNA from control and treated cells were used for double-stranded cDNA synthesis using a High Capacity cDNA Archive kit (Applied Biosystems) according to the manufacturer's instructions. Four differentially expressed genes (*EP3* or *PTGER3*, *EP4* or *PTGER4*, *MMP2* and *MMP9*) were selected for quantitative real-time PCR experiments according to their direct or indirect involvement in inflammatory and metastatic processes based on the Gene Ontology database (http://www.geneonthology.org/). Real-time PCR was performed in triplicate using a 7500 Fast Real-Time PCR System (Applied Biosystems). The reaction mixture consisted of a 20-µl total volume solution containing 10 µl of Power SYBR Green PCR Master Mix (Applied Biosystems), a 1 µM solution of each primer and 20 ng of cDNA. The PCR conditions were 50°C for 2 min and 95°C for 10 min, followed by 40 cycles of 95°C for 15 s and 60°C for 1 min. Melting curve analysis was conducted for each gene to check the specificity and identity of the RT-PCR products. For each primer set, the real-time PCR efficiencies (E) were measured in triplicate on serial dilutions of the same cDNA sample using the formula E = [10(−1/slope)]. The resulting values ranged from 1.96 to 2.02. Each transcript level was normalized to the internal standard *GAPDH*, and the values were Log2-transformed (y-axis) so that all of the values below −1 indicated down-regulation in gene expression, while values above 1 represented up-regulation compared with control cells [43].

### Statistical Analysis

The values concerning the quantification of mast cells and neutrophils of the in vivo tissue samples were expressed as the mean ± SEM of the number of cells per mm^2^ in three sections of 0.5 µm (leaving a space of 40 µm between each section) for each patient (n = 20 patients).

Hep-2 cells were counted using the Countess Automated Cell Counter (Invitrogen). The concentration of the cytokines was determined using MAGPIX Xponent software (Millipore Corporation). The in vitro analyses were repeated a minimum of three times.

The ultrastructural and immunocytochemistry expression of ANXA1 and FPR2 was determined using ten cells for each group investigated. The area of each cell compartment was determined using Axiovision imaging software (Zeiss). In the nucleus and cytoplasm, the density of colloidal gold particles was calculated as the mean ± SEM of the number of particles per µm^2^. In the plasma membrane, the density of colloidal gold particles was calculated as the mean ± SEM of the number of particles per µm.

The significant differences between the means were determined using analysis of variance. This test was followed by the Bonferroni post-hoc test on select experimental groups using Graph-Pad Prism 4.0 (Graph-Pad, San Diego, CA, USA). A probability value less than 0.05 was considered to be statistically significant.

## Results

### The Presence of Degranulated Mast Cells and an Influx of Neutrophils Indicates Inflammation in the Tumor Microenvironment

**Figure 1:**
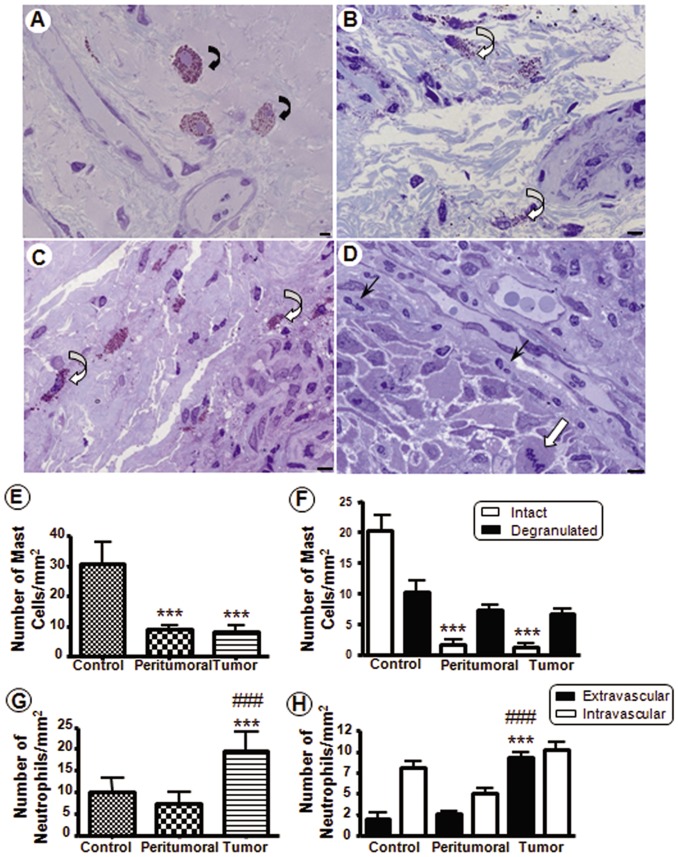
Influx of inflammatory cells in tumor samples from human laryngeal. (**A–D**) Representative images (original magnification, ×63) of Toluidine Blue-stained tissue sections from laryngeal samples. (**A**) Control laryngeal sample showing intact mast cells (curve arrows) near the blood vessels. Peritumoral (**B**) and tumor (**C**) samples with degranulated mast cells (open curve arrows). (**D**) Tumor cells with mitotic activity (open arrow) and inflammatory cells, as extravascular neutrophils (arrows). (**E**) Total number of mast cells. (**F**) Intact and degranulated mast cells. (**G**) Total number of neutrophils. (**H**) Extra and intravascular neutrophils. Data are expressed as the mean ± SEM of the cell number per mm^2^ (n = 20 patients per group). One-way ANOVA followed by Bonferroni's test revealed a significant difference among the groups. *** *P*<0.001 *vs.* control, ### *P*<0.001 *vs.* peritumoral. Scale bars: 5 µm.

To verify the interaction of inflammatory cells with laryngeal tumor cells, we performed histological analyses in larynx tissue samples to quantify mast cells and neutrophils, important cells of the inflammatory response. In control samples, we observed intact mast cells with metachromatic granules in close vicinity to blood vessels (Fig. 1A). In the peritumoral and tumor stroma, we verified the presence of degranulated mast cells (Fig. 1, B and C). In addition, we observed neutrophils that had transmigrated into tumor sections (Fig. 1D).

Statistical analysis showed that there were high numbers of mast cells in the control sections of the larynx and significantly fewer mast cells in the peritumoral and tumor regions (*P*<0.001; Fig. 1E). Furthermore, intact mast cells were more abundant in control samples and significantly lower in peritumoral and tumor sections (*P*<0.001; Fig. 1F). Using quantitative analysis of the neutrophils, we verified that there was a significant increase in these cells in the tumor tissues (*P*<0.001) compared with control and peritumoral tissues (Fig. 1G). Examination of the neutrophils in control and peritumoral sections showed that most of these cells were localized in the blood vessels (Fig. 1H). By contrast, in the tumor samples, we observed a significant number of transmigrated neutrophils in the stroma (*P*<0.001 compared with control and peritumoral samples) (Fig. 1H).

### Up-Regulation of ANXA1/FPR2 in the Inflammatory Cells of Human Laryngeal Tumors

**Figure 2:**
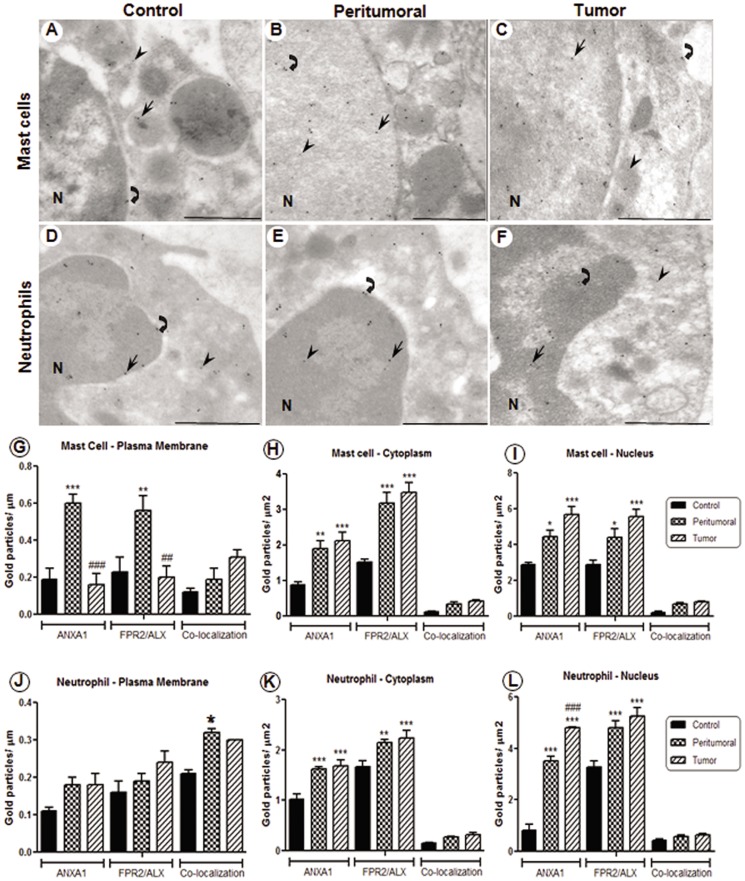
Immunoreactivity to ANXA1 and FPR2/ALX in mast cells and neutrophils of laryngeal sections. Immunolabeling with 10-nm (FPR2/ALX) and 15-nm (ANXA1) colloidal gold particles. Mast cells (**A–C**) and neutrophils (**D–F**) showing subcellular localization of ANXA1 (arrows) and FPR2/ALX (arrowheads), and co-localization (curve arrows) in the plasma membrane, cytoplasm and nucleus (N) of control, peritumoral and tumor tissues. Density of gold particles conjugated with ANXA1 and FPR2/ALX in mast cells (**G–I**) and neutrophils (**J–L**). Data are expressed as the mean ± SEM of colloidal gold particles per µm in the plasma membrane and per µm^2^ in the cytoplasm and nucleus of cells (n = 10 cells per group). One-way ANOVA followed by Bonferroni's test revealed a significant difference among the groups. * *P*<0.05, ** *P*<0.01, *** *P*<0.001 *vs.* control, ## *P*<0.01, ### *P*<0.001 *vs.* peritumoral. Stained with uranyl acetate and lead citrate. Scale bars: 1 µm.

Ultrastructural immunocytochemistry showed for the first time the expression of FPR2/ALX and its co-localization with ANXA1 in mast cells (Fig. 2, A–C). The mast cells and neutrophils displayed ANXA1 and FPR2/ALX immunoreactivity in the plasma membrane, cytoplasm and nucleus (Fig. 2, A–F). No immunogold labeling was detected in sections that were incubated with nonimmune serum (data not shown). In these inflammatory cells, we verified a significant up-regulation of ANXA1 and FPR2/ALX expression in the peritumoral and tumor samples compared with the corresponding control tissues (Fig. 2, G–L).

Quantitative analysis revealed that there is higher expression of ANXA1 and its receptor in the cytoplasm and nucleus compared with that in the plasma membrane (Fig. 2, G–L). Co-localization of ANXA1 and FPR2/ALX was observed in the plasma membrane, cytoplasm and nucleus of mast cells and neutrophils (Fig. 2, G–L); however there was significant difference only in plasma membrane of peritumoral neutrophils (Fig. 2J).

### ANXA1_2–26_ Inhibits FPR2/ALX Receptor-Mediated Proliferation of Hep-2 Laryngeal Cancer Cells

**Figure 3:**
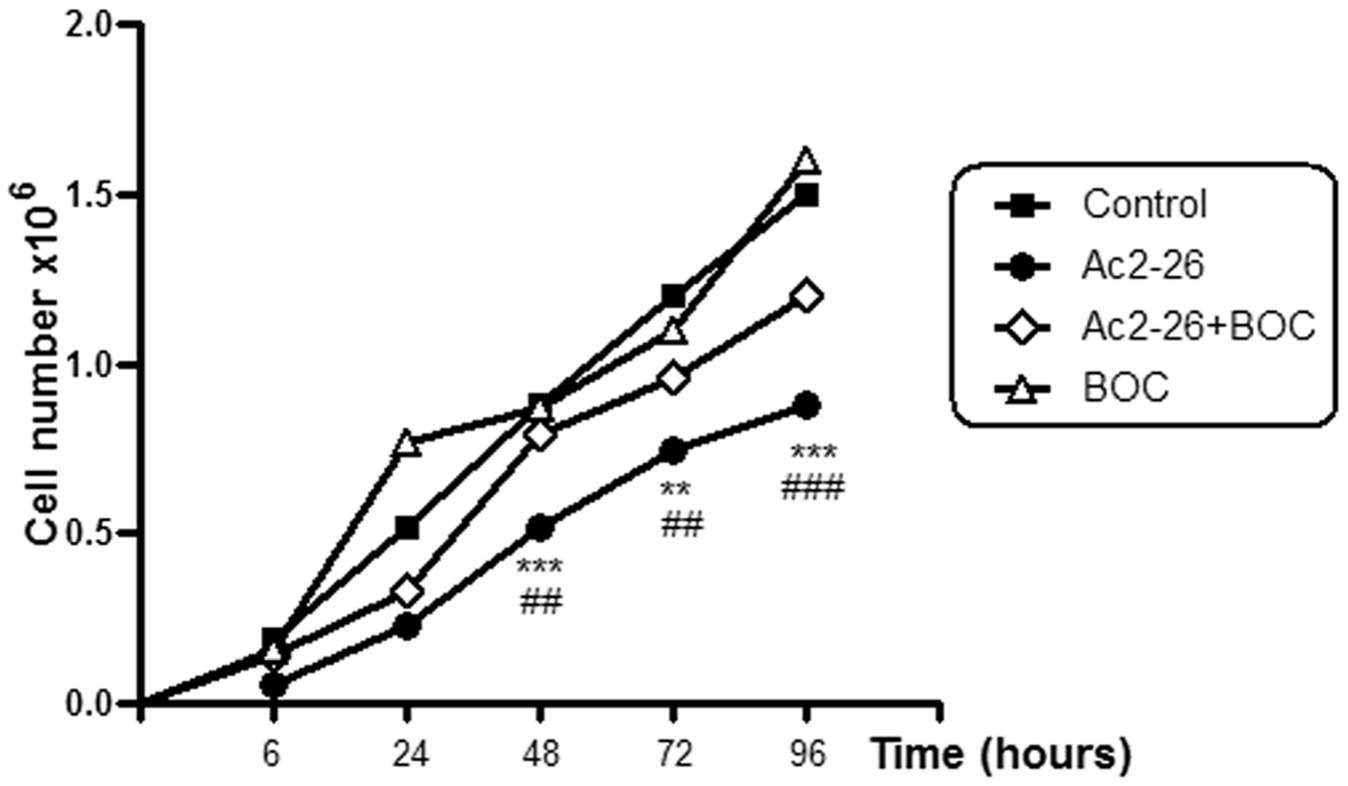
Effect of the peptide ANXA1_2–26_ and antagonist Boc2 on the proliferation of Hep-2 cells. Treatment with ANXA1_2–26_ reduced the cellular growth. The antagonist Boc2 partially inhibited the anti-proliferative effect of ANXA1_2–26_. The cells treated with Boc2 alone showed a level of proliferation similar to that of the control. The Hep-2 cells were seeded in MEM-Earle medium at a density of 2×10^6^ cells in 75-cm^2^ culture flask, and then were incubated with serum-free medium 24 hours prior to the addition of ANXA1_2–26_ (1 µM), ANXA1_2–26_ (1 µM)+Boc (10 µM) or Boc (10 µM) alone. All of the experiments were performed in triplicate to confirm the results. Data are expressed as the mean ± SEM of the cell number ×10^6^. ** *P*<0.01 and *** *P*<0.001 *vs.* control, ## *P*<0.01 and ### *P*<0.001 *vs.* ANXA1_2–26_+Boc.

Approximately 87% to 97% of the Hep-2 cells were viable under the experimental conditions (control, ANXA1_2–26_, ANXA1_2–26_+Boc2, Dexa, Dexa+Boc2 and Boc2) (S1 Figure). However, the growth curve of these cells showed significant differences among the conditions studied (Fig. 3).

Treatment with the peptide ANXA1_2–26_ significantly reduced the proliferation of Hep-2 laryngeal cancer cells compared with the control cells (*P*<0.001 at 48 and 96 hours, and *P*<0.01 at 72 hours). Similar results were obtained after treatment with Dexa and Dexa+Boc2 (S2 Figure). However, combination treatment with ANXA1_2–26_ and Boc2 (ANXA1_2–26_+Boc) significantly augmented cell growth compared with ANXA1_2–26_-treated cells (*P*<0.01 at 48 and 72 hours, and *P*<0.001 at 96 hours), demonstrating that Boc2 attenuated the antiproliferative activity of ANXA1_2–26_. Boc2-treated cells had a proliferative rate that was similar to that of control cells (Fig. 3).

### Loss of ANXA1/FPR2 Protein in Laryngeal Tumor Tissues and Up-regulation after *In Vitro* Treatment with the ANXA1_2–26_ Peptide

**Figure 4:**
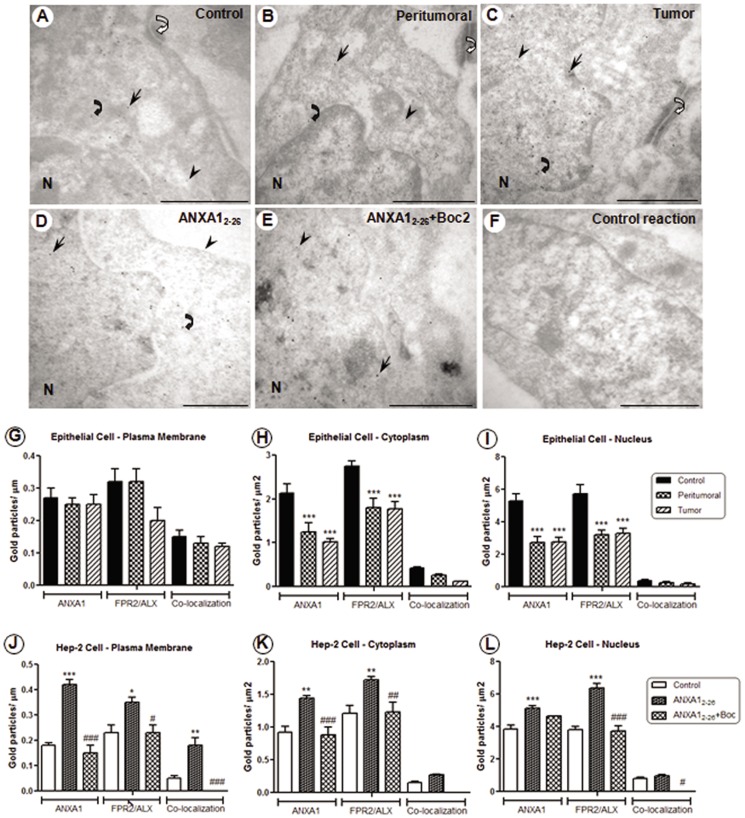
Ultrastructural immunocytochemistry detection of ANXA1 and FPR2/ALX co-localization in laryngeal cells by electron microscopy. Immunolabeling with 10-nm (FPR2/ALX) and 15-nm (ANXA1) colloidal gold particles. (**A–C**) Epithelial control, peritumoral and tumor cells from laryngeal tissues. (**D–E**) Hep-2 cells showing immunoreactivity to ANXA1 (arrows), FPR2 (arrowheads), and co-localizations (curve arrows) in the plasma membrane, cytoplasm and nucleus (N). Desmosomes (open curve arrows) are detected between these cells. (**F**) The absence of immunoreactivity to ANXA1 and FPR2/ALX in cells incubated with nonimmune serum. Density of gold particles conjugated with ANXA1 and FPR2/ALX in epithelial cells (**G–I**) and Hep-2 cells (**J–L**). Hep-2 cells were seeded in MEM-Earle medium at a density of 2×10^6^ cells in 75-cm^2^ culture flasks, and then were incubated with serum-free medium 24 hours prior to the addition of ANXA1_2–26_ (1 µM) and ANXA1_2–26_ (1 µM)+Boc2 (10 µM). Data are expressed as the mean ± SEM of colloidal gold particles per µm in the plasma membrane and per µm^2^ in the cytoplasm and nucleus of cells (n = 10 cells per group). One-way ANOVA followed by Bonferroni's test revealed a significant difference among the groups. * *P*<0.05, ** *P*<0.01, *** *P*<0.001 *vs.* control, # *P*<0.05, ## *P*<0.01, ### *P*<0.001 *vs.* ANXA1_2–26_. Stained with uranyl acetate and lead citrate. Scale bars: 1 µm.

The epithelial cells of control, peritumoral and tumor laryngeal tissues displayed immunoreactivity to ANXA1 and FPR2/ALX, as well as co-localizations of the proteins, in the plasma membrane, cytoplasm and nucleus (Fig. 4, A–C). In the peritumoral and tumor samples, we observed a marked reduction of ANXA1 and FPR2/ALX protein expression compared with the corresponding control tissues (Fig. 4, H and I). Quantitative analysis revealed that there was higher expression of ANXA1 and its receptor in the cytoplasm and nucleus and lower expression in the plasma membrane (Fig. 4, G–I). Differences in the degree of ANXA1/FPR2 co-localization did not reach statistical significance among the laryngeal samples (Fig. 4, G–I).

Hep-2 cells that were treated with ANXA1_2–26_ or ANXA1_2–26_ plus Boc2 showed immunoreactivity for ANXA1 and FPR2/ALX (Fig. 4, D and E). Co-localization of the two proteins was detected in the plasma membrane, cytoplasm and nucleus of these cells but not in Boc2-treated cells (Fig. 4, D and E). No gold labeling was detected in cells that were incubated with nonimmune serum (Fig. 4 F). ANXA1/FPR2 expression was down-regulated in the plasma membrane but was increased in the cytoplasm and nucleus of Hep-2 cells (Fig. 4, J–L). Treatment with ANXA1_2–26_ displayed up-regulation of ANXA1 and FPR2 in the cytoplasm and nucleus compared with the corresponding control cells (Fig. 4, J–L). Furthermore, we detected marked reduction of ANXA1 protein and its receptor after ANXA1_2–26_+Boc2 treatment compared with ANXA1_2–26_-treated cells (Fig. 4, J–L). Treatment with ANXA1_2–26_ significantly augmented the degree of ANXA1/FPR2 co-localization in the plasma membrane of Hep-2 cells (Fig. 4 J).

### Decreased Pro-inflammatory Cytokine Levels Characterizes the Anti-inflammatory Effect of ANXA1 on Laryngeal Cancer Cells

**Figure 5:**
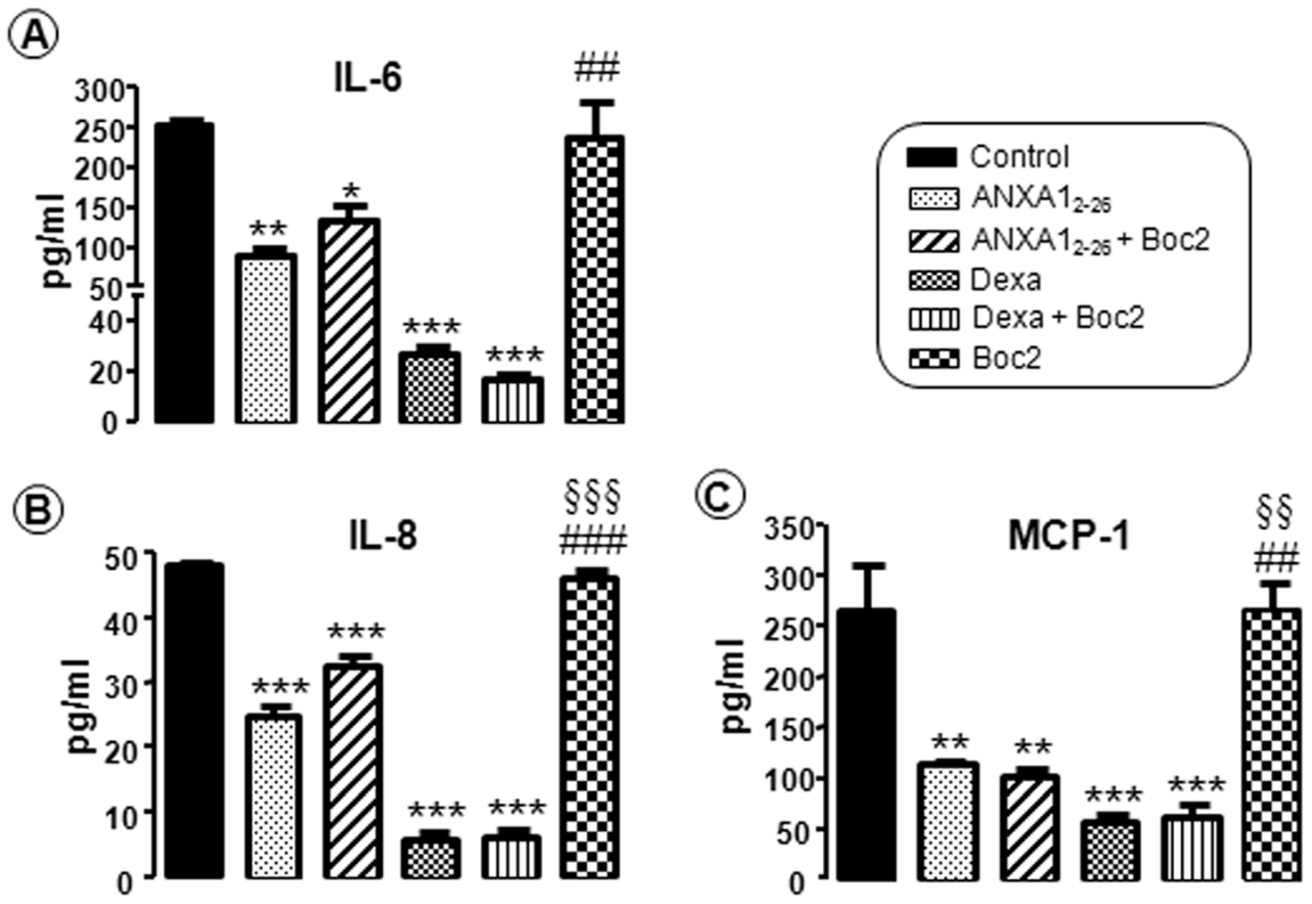
Effect of the peptide ANXA1_2–26_ on proinflammatory cytokine expression. Low expression of IL-6 (**A**), IL-8 (**B**) and MCP-1 (**C**) after treatment with ANXA1_2–26_ and dexamethasone. Hep-2 cells were seeded in MEM-Earle medium at a density of 2×10^6^ cells in 75-cm^2^ culture flasks, and then were incubated with serum-free medium 24 hours prior to the addition of ANXA1_2–26_ (1 µM), ANXA1_2–26_ (1 µM)+Boc2 (10 µM), Dexa (0.01 µM), Dexa (0.01 µM)+Boc2 (10 µM) or Boc2 (10 µM) alone. All of the experiments were performed in triplicate to confirm the results. Data are expressed as the mean ± SEM of the analyte concentration (pg/mL), determined using *MAGPIX xPONENT software*. * *P*<0.05, ** *P*<0.01 and *** *P*<0.001 *vs.* control, ## *P*<0.01 and ### *P*<0.001 *vs.* ANXA1_2–26_, §§ *P*<0.01 and §§§ *P*<0.001 *vs.* ANXA1_2–26_+Boc2.

The expression pattern of IL-6, IL-8 and MCP-1 was similar among the experimental conditions (Fig. 5, A–C). The Hep-2 cells that were incubated with ANXA1_2–26_, ANXA1_2–26_+Boc2, Dexa and Dexa+Boc2 showed a marked reduction in cytokine levels. However, the Boc2-treated cells showed a high level of cytokine expression that was similar to that observed under control conditions (Fig. 5, A–C).

### EP3- and EP4-Mediated Metalloproteinase Signaling Can Be Regulated by ANXA1_2–26_ in Tumor Cells

To investigate the possible effect of ANXA1 on the expression of genes that are involved in the prostaglandin pathway and, therefore, are related to tumorigenesis and inflammation, we carried out real time PCR in Hep-2 cells that were incubated with ANXA1_2–26_ and Boc2. Prior to RT-PCR, we evaluated the quality of mRNA and cDNA obtained from the samples (S3 Figure, A and B).

**Figure 6:**
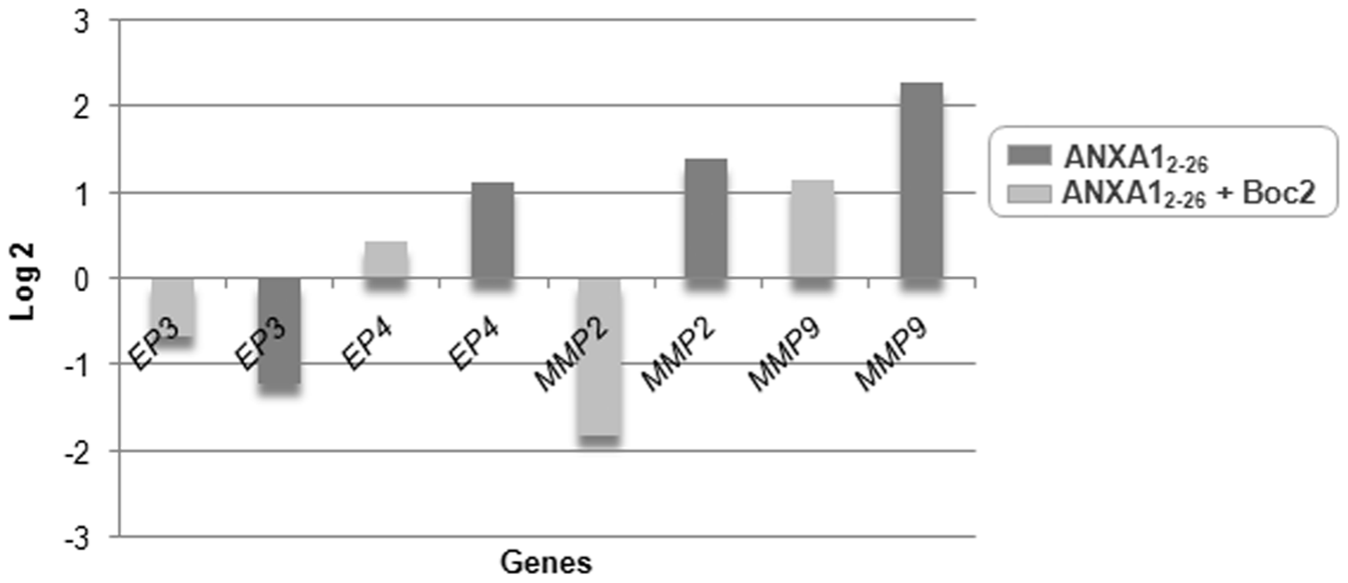
Gene expression in Hep-2 cells using real-time PCR. (**A**) Down- and up-regulated expression of *MMP2* and *MMP9*, respectively, after ANXA1_2–26_ treatment. (**B**) Overexpression of *EP4*, *MMP2* and *MMP9*, and down-regulation of *EP3* after ANXA1_2–26_+Boc. Hep-2 cells were seeded in MEM-Earle medium at a density of 2×10^6^ cells in 75-cm^2^ culture flasks, and then were incubated with serum-free medium, 24 hours prior to the addition of ANXA1_2–26_ (1 µM) and ANXA1_2–26_ (1 µM)+Boc2 (10 µM). All of the experiments are representative of three separate experiments. *GAPDH* was used as the internal reference and the control sample was used as the calibrator. Values were Log2 transformed (y-axis) so that all values below −1 indicated down-regulation in gene expression while values above 1 represented up-regulation compared with control cells.

Hep-2 cells that were treated with ANXA1_2–26_ showed a significant decrease (Log2≤−1.0) in *MMP2* expression and an increase (Log2≥1.0) in *MMP9* expression compared with control cells (Fig. 6). However, incubation of Hep-2 cells with ANXA1_2–26_ plus Boc mediated up-regulation (Log2≥1.0) of *EP4*, *MMP2* and *MMP9* gene expression and down-regulation (Log2≤−1.0) of *EP3* expression compared with control cells (Fig. 6).

## Discussion

Although advances in new diagnostic tools and treatments have led to declining mortality rates, cancer remains a leading cause of death in industrialized countries. A better understanding of the interactions between tumors and inflammation may lead to new biological targets for therapeutic intervention in different neoplasms within the context of chronic inflammation, such as laryngeal carcinoma. We showed that ANXA1 protein may control tumor growth in a paracrine manner that is mediated by the receptor FPR2/ALX. In the inflammatory cells of human laryngeal carcinoma tissue samples, ANXA1/FPR2 expression was markedly exacerbated; however, in laryngeal carcinoma cells, this expression was down-regulated. Treatment with ANXA1_2–26_ reduced the proliferation of Hep-2 cells and up-regulated ANXA1/FPR2 expression in these cells. Our results established, for the first time, the role of FPR2/ALX in laryngeal carcinoma cells as demonstrated by ultrastructural analyses showing the co-localization of ANXA1/FPR2.

The inflammatory components in a developing neoplasm may include several types of immune cells, including mast cells and neutrophils, which can produce an array of cytokines, such as the cell-killing mediators TNF-α and interleukins (ILs) [48], [49]. The presence of degranulated mast cells in the peritumoral and tumor laryngeal tissues suggests that neoplastic cells may recruit and activate mast cells to release mediators that could either be detrimental to the tumor or contribute to its development [50]. Intriguingly, most reports show the localization of mast cells predominantly at the tumor periphery [51] and associated with the vasculature surrounding malignant tissues, suggesting that these cells play a pro-angiogenic role [52]. The scarcity of mast cells within the tumor core may be a histology artifact that results from mast cell degranulation, leading to “ghosts” following staining [53]. Our data indicate that mast cells may represent a possible target for therapeutic intervention in controlling tumor growth and angiogenesis in laryngeal carcinoma.

Mast cells may be effective in inducing neutrophil migration to inflammatory sites [54], numerous transmigrated neutrophils were observed in the larynx tumor samples. Despite their known function as phagocytes, the role of tumor-associated neutrophils in neoplastic progression has been controversial [19], [55]. Neutrophils can induce DNA damage in proliferating cells through the generation of reactive oxygen and nitrogen species, which are normally produced by these cells to fight infection [55]. However, neutrophils can have both tumor-promoting and tumoricidal functions, depending on the presence of TGF-β [20]. The recognition of neutrophils as a key component of tumor growth opens an avenue for novel approaches to cancer therapies to decrease laryngeal carcinoma cell proliferation and metastasis in patients.

It is now evident that inflammatory cells have powerful effects on tumor development. Early in the neoplastic process, these cells are malignant promoters, producing an attractive environment for tumor growth, facilitating genomic instability and promoting angiogenesis [21]. Inflammatory cells can regulate the growth, migration and differentiation of all cell types in the tumor microenvironment [49]. Thus, it is clear that anti-inflammatory therapy is efficacious toward preventing early neoplastic progression.

In this anti-inflammatory and anti-proliferative study, we have unveiled whether ANXA1 protein could be mediated by receptors for formylated peptides (FPRs) in larynx cancer. The functions of FPRs in cancer are still not well established.

We analyzed the role of ANXA1/FPR2 interactions in the proliferation of Hep-2 cells after treatment with ANXA1_2–26_, dexamethasone and/or Boc2. The addition of ANXA1_2–26_ and Dexa to the cell culture reduced cell growth, showing the antiproliferative effects of these anti-inflammatory drugs. However, the pharmacological antagonist Boc2 attenuated the antiproliferative activity of ANXA1, suggesting that this receptor plays central roles in the proliferation response in larynx cancer. These data corroborate the results of a recent study [56], which showed that the effects of ANXA1 on breast tumor cell proliferation were prevented by Boc2.

To uncover the subcellular localization and possible interaction of ANXA1/FPR2, we performed immunocytochemistry reactions in the inflammatory and epithelial cells of laryngeal tissue samples and in Hep-2 cells. Co-localization of ANXA1/FPR2 was detected in the mast cells, neutrophils, and tumor cells. These data suggest that the anti-inflammatory/antiproliferative activity of ANXA1 may be mediated by FPR2/ALX and show, for the first time, the presence of the receptor FPR2/ALX and its interaction with ANXA1 in mast cells and laryngeal tumor cells. Recently, we also detected the co-localization of ANXA1/Fpr2 in murine neutrophils, suggesting that the anti-inflammatory ANXA1 effects may be mediated through this receptor [57].

The mast cells and neutrophils in the peritumoral and tumor sections revealed high expression of ANXA1/FPR2. The tumor-activated inflammatory cells appear to increase the synthesis of ANXA1/FPR2 proteins as an anti-inflammatory response mechanism to resolve the inflammation and proliferation in laryngeal cancer. The cellular differentiation and, in some cases, activation are stimuli for the synthesis of ANXA1, although the molecular processes are not yet fully understood [58].

Loss of ANXA1 and FPR2/ALX expression was detected in laryngeal carcinoma cells. However, Hep-2 cells had up-regulated expression of ANXA1, FPR2/ALX and co-localization after treatment with ANXA1_2–26_ peptide. These results could signify the regulatory role of the ANXA1/FPR2 interaction in cancer development and, corroborate the previous investigation performed in our laboratory [32] demonstrating that ANXA1_2–26_-treated Hep-2 cells exhibited increased ANXA1 expression as shown by real time PCR. In vitro treatment with ANXA1_2–26_+Boc2 markedly reduced the expression of ANXA1/FPR2 in Hep-2 cells. This result confirms that the antagonist Boc2 blocks the antiproliferative FPR2-mediated activity of ANXA1. Overexpression of ANXA1 protein is observed in hepatocellular carcinoma [29] and pancreatic cancer [59]. By contrast, reduced levels of ANXA1 protein expression have been reported in esophageal cancer [30] and laryngeal cancer [32]. The discrepancy in these studies indicates that the effects of ANXA1 on cancer cells may be cell-type specific and that factors other than the expression level may affect its function.

The electron microscopy data demonstrated that ANXA1/FPR2 expression was high in the cytoplasm and nucleus and low in the plasma membrane. ANXA1, which is usually located in the cytoplasm, can translocate to the nucleus after stimulation. Recently, Lin and colleagues [60] analyzed 115 patients with oral carcinoma and observed high expression of ANXA1 in the nucleus of epithelial tumor cells that correlated with poor prognosis. Although these authors [60] have shown that ANXA1 translocation to the nucleus correlates with the regulation of epithelial cell proliferation and may represent a candidate prognostic marker [43], the precise physiological role of ANXA1 in the nucleus remains poorly understood.

Because of the importance of cytokine control of the inflammatory microenvironment favoring or inhibiting tumor progression [48], [61], we investigated the level of IL-6, IL-8 and MCP-1 released by Hep-2 cells. IL-6 is produced by tumor and stromal cells and contributes to tumorigenesis by various mechanisms, including angiogenesis, cell survival and metastasis [62]. IL-6 signaling leads to the increased production of inflammatory cytokines, proteases and growth factors, such as IL-1β, IL-8, MCP-1, MMP9, MMP2 and VEGF [61], [63]. Our data showed that ANXA1 and dexamethasone inhibit the release of inflammatory cytokines by Hep-2 cells, which may prevent the development of laryngeal squamous cell carcinoma.

Additionally, we have begun to outline the anti-inflammatory pathway of ANXA1 in Hep-2 cells which may result in the inhibition of angiogenic and invasive phenotypes. Thus, we investigated the effect of ANXA1 on the expression of genes that are involved in prostaglandin signaling and whose role in head and neck cancer deserves evaluation [41]: *EP3 and EP4* and their downstream effectors *MMP2* and *MMP9*. Prostaglandins are lipid mediators of inflammation released in response to different stimuli, including cigarette smoking [64]. They are synthesized from arachidonic acid by cyclooxygenase (COX) in many cells, such as mast cells, and exert their signaling effects through G-protein-coupled receptors (reviewed by Aoki and Narumiya) [65].

Prostaglandin E2 is the most important COX metabolite present in human tumors and that bind to its receptors (EP1–4) on the plasma membrane of neoplastic, stromal or immune cells. The EP receptors may have distinct functions depending on the cell and tissue type [66]. For example, EP4 is linked to cAMP/PKA and PI3K/Akt pathways whereas the multiple isoforms of EP3 may negatively or positively regulate cAMP [67]. They can transactivate the epidermal growth factor receptor (EGFR), a biomarker that is virtually upregulated in all head and neck tumors, inducing the expression of several proteins, including COX 2, matrix metalloproteinases 2 and 9 and vascular endothelial growth factor [68]. Otherwise, EP signaling may stimulate EGFR ligand release via matrix metalloproteinases [69]. These molecular signaling cascades promote tumor cell proliferation, angiogenesis and extracellular matrix degradation and may be targets for the treatment and prevention of cancer. Indeed, several epidemiological studies have shown an inverse correlation between cancer risk and non-steroidal anti-inflammatory drugs, which are potent inhibitors of cyclooxygenase (COX) [68]. Treatment of Hep-2 cells with ANXA1_2–26_ reduced *MMP2* expression and increased *MMP9* expression. Unexpectedly, up-regulation of *EP4*, *MMP2* and *MMP9* and down-regulation of *EP3* expression were observed after treatment with ANXA1_2–26_ and Boc2. Te latter findings show that ANXA1 may affect the expression of MMPs and EP receptors through its interaction with the receptor FPR2/ALX. Therefore, may interact with different receptors, generating signals that are interconnected with the prostaglandin pathway. Clearly, further investigation is needed.

Overall, our in vivo data indicated that mast cells and neutrophils, under activation by the tumor microenvironment, demonstrate up-regulation of ANXA1/FPR2 as an anti-inflammatory response mechanism to resolve inflammation and proliferation in laryngeal cancer. However, in vitro results have indicated that treatment with the peptide mimetic ANXA1_2–26_ promotes the reduction of Hep-2 cell proliferation, up-regulation of ANXA1/FPR2 expression, down-regulation of inflammatory cytokines (IL-6, IL-8 and MCP-1) and MMP2.

## Conclusions

Integration of the in vivo and in vitro data showed that ANXA1 may be effective in the regulation of tumor growth and metastasis through paracrine mechanisms mediated by FPR2/ALX. Detailed studies of mast cell and neutrophil function in different types of cancer should aid the development of effective antitumor strategies. In light of the potential importance ascribed to ANXA1, our study not only has generated novel and important information about ANXA1 mechanisms but also may justify further investigation of the roles of the complex FPR receptor family. In addition, a combination of anti-inflammatory approaches that target the tumor microenvironment with more sophisticated and selective tumoricidal drugs may lead to future therapeutic intervention in human laryngeal cancer.

## Supporting Information

S1 Figure Analysis of Hep-2 cell viability. Treatment with ANXA1_2–26_, ANXA1_2–26_+Boc2, Dexa, Dexa+Boc2 or Boc2 did not affect the percentage of cellular viability during growth curve analysis. The Hep-2 cells were seeded in MEM-Earle medium at a density of 2×10^6^ cells in 75-cm^2^ culture flasks, and then were incubated with serum-free medium, 24 hours prior to the addition of ANXA1_2–26_ (1 µM), ANXA1_2–26_ (1 µM)+Boc2 (10 µM), Dexa (0.01 µM), Dexa (0.01 µM)+Boc2 (10 µM) or Boc2 (10 µM) alone. All experiments were performed in triplicate to confirm the results. Data are expressed as the mean ± SEM of the cell percentage number.
(TIF)

S2 Figure Effect of dexamethasone on the proliferation of Hep-2 cells. Treatment with dexamethasone (Dexa) reduced the cellular growth. The antagonist Boc2 had no effect on Dexa. Hep-2 cells were seeded in MEM-Earle medium at a density of 2×10^6^ cells in 75-cm^2^ culture flasks, and then were incubated with serum-free medium, 24 hours prior to the addition of Dexa (0.01 µM) and Dexa (0.01 µM)+Boc2 (10 µM). All of the experiments were performed in triplicate to confirm the results. Data are expressed as the mean ± SEM of the cell number ×10^6^. ** *P*<0.01, *** *P*<0.001 *vs.* control.
(TIF)

S3 Figure Validation of mRNA and cDNA integrity. Agarose gels showing the quality of mRNA (A) and cDNA (B) from Hep-2 cells after treatment. Hep-2 cells were seeded in MEM-Earle medium at a density of 2×10^6^ cells in 75-cm^2^ culture flasks, and then were incubated with serum-free medium, 24 hours prior to the addition of ANXA1_2–26_ (1 µM) and ANXA1_2–26_ (1 µM)+Boc2 (10 µM). All of the experiments were performed in triplicate to confirm the results.
(TIF)
