# Supplementary figures and images for: Inflammation and Cancer: Role of Annexin A1 and FPR2/ALX in Proliferation and Metastasis in Human Laryngeal Squamous Cell Carcinoma
Source: PLoS One. 2014 Dec 9;9(12):e111317. doi: 10.1371/journal.pone.0111317 (PMC4260827; doi:10.1371/journal.pone.0111317)

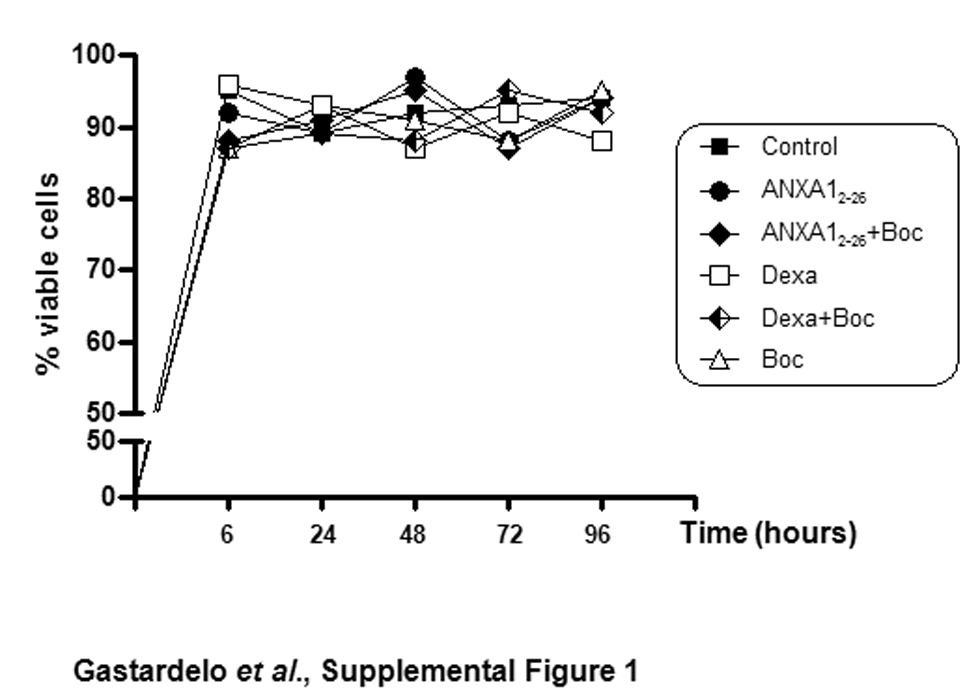

Supplement: S1 Figure — Analysis of Hep-2 cell viability. Treatment with ANXA12–26, ANXA12–26+Boc2, Dexa, Dexa+Boc2 or Boc2 did not affect the percentage of cellular viability during growth curve analysis. The Hep-2 cells were seeded in MEM-Earle medium at a density of 2×10^6^ cells in 75-cm2 culture flasks, and then were incubated with serum-free medium, 24 hours prior to the addition of ANXA12–26 (1 µM), ANXA12–26 (1 µM)+Boc2 (10 µM), Dexa (0.01 µM), Dexa (0.01 µM)+Boc2 (10 µM) or Boc2 (10 µM) alone. All experiments were performed in triplicate to confirm the results. Data are expressed as the mean ± SEM of the cell percentage number. (TIF) [file pone.0111317.s001.tif]

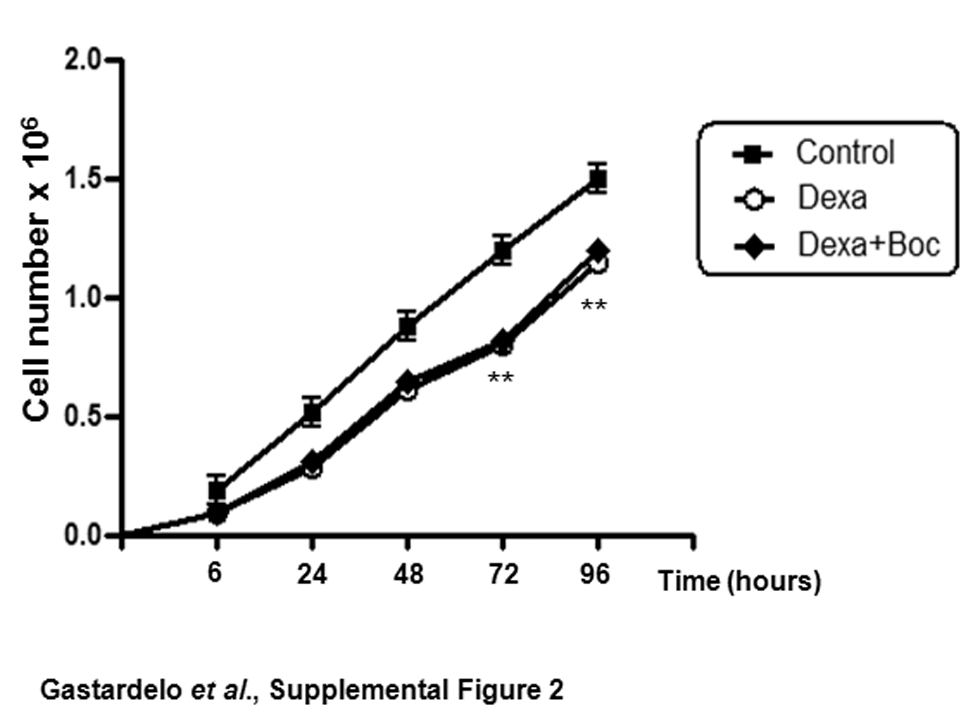

Supplement: S2 Figure — Effect of dexamethasone on the proliferation of Hep-2 cells. Treatment with dexamethasone (Dexa) reduced the cellular growth. The antagonist Boc2 had no effect on Dexa. Hep-2 cells were seeded in MEM-Earle medium at a density of 2×10^6^ cells in 75-cm2 culture flasks, and then were incubated with serum-free medium, 24 hours prior to the addition of Dexa (0.01 µM) and Dexa (0.01 µM)+Boc2 (10 µM). All of the experiments were performed in triplicate to confirm the results. Data are expressed as the mean ± SEM of the cell number ×106. ** P<0.01, *** P<0.001 vs. control. (TIF) [file pone.0111317.s002.tif]

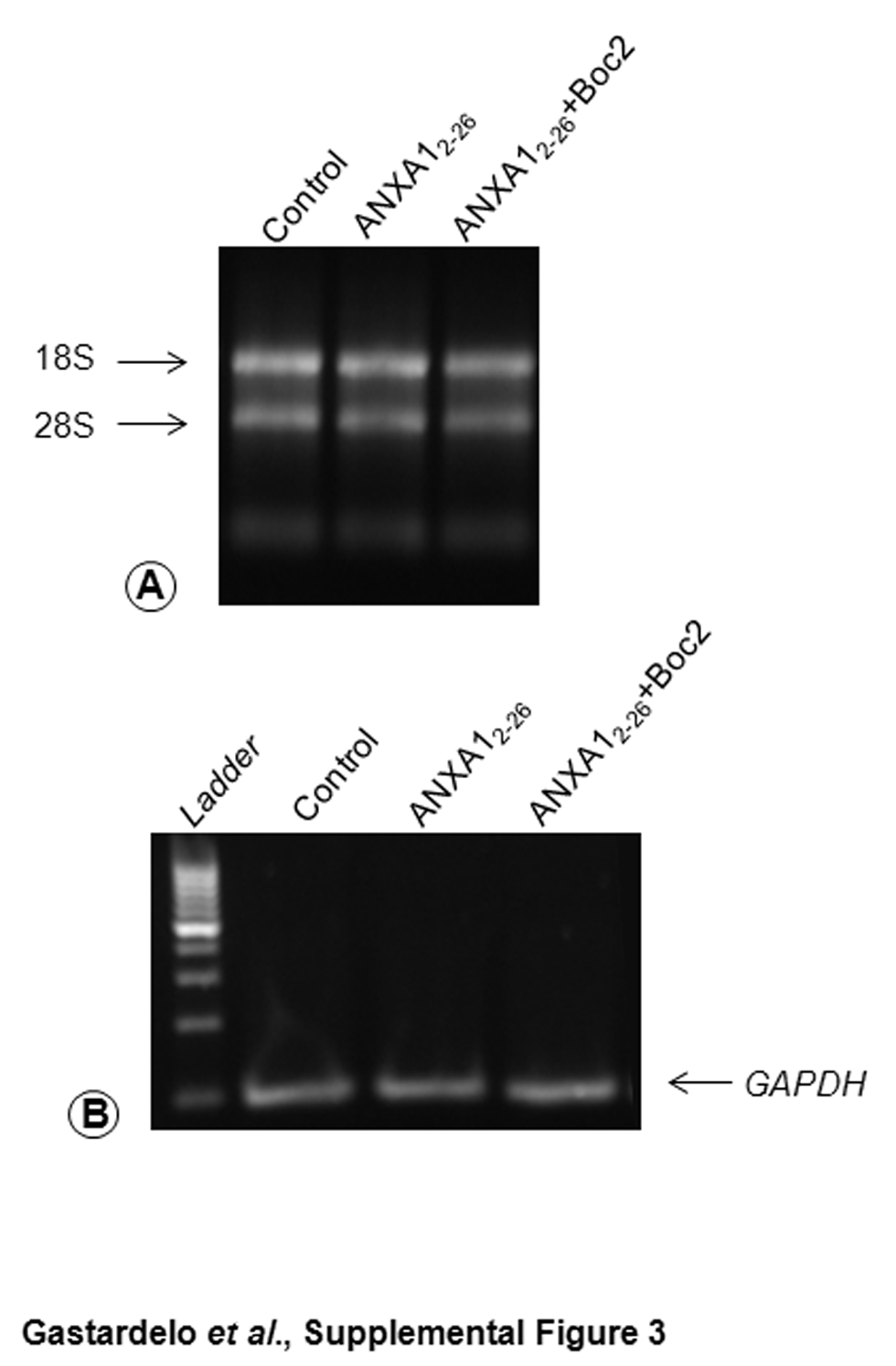

Supplement: S3 Figure — Validation of mRNA and cDNA integrity. Agarose gels showing the quality of mRNA (A) and cDNA (B) from Hep-2 cells after treatment. Hep-2 cells were seeded in MEM-Earle medium at a density of 2×10^6^ cells in 75-cm2 culture flasks, and then were incubated with serum-free medium, 24 hours prior to the addition of ANXA12–26 (1 µM) and ANXA12–26 (1 µM)+Boc2 (10 µM). All of the experiments were performed in triplicate to confirm the results. (TIF) [file pone.0111317.s003.tif]
